# Prying into the intimate secrets of animal lives; software beyond hardware for comprehensive annotation in ‘Daily Diary’ tags

**DOI:** 10.1186/s40462-015-0056-3

**Published:** 2015-09-21

**Authors:** James S. Walker, Mark W. Jones, Robert S. Laramee, Mark D. Holton, Emily LC Shepard, Hannah J. Williams, D. Michael Scantlebury, Nikki, J. Marks, Elizabeth A. Magowan, Iain E. Maguire, Owen R. Bidder, Agustina Di Virgilio, Rory P. Wilson

**Affiliations:** Department of Computer Science, College of Science, Swansea University, Singleton Park, Swansea, SA2 8PP, Wales UK; College of Engineering, Swansea University, Singleton Park, Swansea, SA2 8PP, Wales UK; Swansea Lab for Animal Movement, Biosciences, College of Science, Swansea University, Singleton Park, Swansea, SA2 8PP, Wales UK; School of Biological Sciences, Institute for Global Food Security, Queen’s University Belfast, Belfast, BT9 7BL, Northern Ireland UK; Institut für Terrestrische und Aquatische Wildtierforschung, Stiftung Tierärztliche Hochschule, Werfstr. 6, 25761, Hannover, Büsum Germany; Laboratorio Ecotono, INIBIOMA-CONICET, National University of Comahue, Quintral 1250, (8400), Bariloche, Argentina

## Abstract

**Background:**

Smart tags attached to freely-roaming animals recording multiple parameters at infra-second rates are becoming commonplace, and are transforming our understanding of the way wild animals behave. Interpretation of such data is complex and currently limits the ability of biologists to realise the value of their recorded information.

**Description:**

This work presents Framework4, an all-encompassing software suite which operates on smart sensor data to determine the 4 key elements considered pivotal for movement analysis from such tags (Endangered Species Res 4: 123-37, 2008). These are; animal trajectory, behaviour, energy expenditure and quantification of the environment in which the animal moves. The program transforms smart sensor data into dead-reckoned movements, template-matched behaviours, dynamic body acceleration-derived energetics and position-linked environmental data before outputting it all into a single file. Biologists are thus left with a single data set where animal actions and environmental conditions can be linked across time and space.

**Conclusions:**

Framework4 is a user-friendly software that assists biologists in elucidating 4 key aspects of wild animal ecology using data derived from tags with multiple sensors recording at high rates. Its use should enhance the ability of biologists to derive meaningful data rapidly from complex data.

## Background

The development of hardware that can be attached to animals during their everyday life [31] has revolutionized our understanding of the biology of wild animals. Indeed, this approach has allowed researchers to look at everything from the behaviour of whales chasing prey at depths of over 1 km underwater [1] to the physiology of geese migrating over the Himalayas [18]. A common feature facilitating these sorts of projects has been the increase in numbers and types of sensors used in smart animal tags, as well as increases in the frequency with which they can be sampled and concomitant increases in memory capacity. Thus, our ability to answer critical questions in biology appears to have been driven to a large extent by advances in technology [31]. These advances in methodology come under two broad areas. One relates to methods that allow tags to be physically attached to animals for increasing lengths of time (e.g. [19, 32, 46]) while minimizing animal detriment [37, 38] while the other relates to the physical production of the complex solid-state units in smart tags (e.g. [24]).

The excitement at the potential inherent in sophisticated animal tags has, however, been tempered by a new limiting factor. This is a methodology to deal with the problem of the analysis of the high resolution, multiple channel (and therefore multi-dimensional) data acquired by the tags – in short, software (cf. [22]). To be most useful to the community, software to help in the analysis of smart tag-acquired data needs to be able to deal with large quantities of multiple sensor data and, ideally, should be able to merge different derived analytical outputs together into one output file so that various elements derived from the primary data can be interrelated. Currently, the smart tag community has witnessed a number of software innovations, most of which are concerned with determination of behaviour, i.e. from dive profile data [15] or, most notably, from dual-axial acceleration data [33, 41]. Analysis of acceleration data is particularly welcome because inspection of raw acceleration values over time to derive behaviours is not particularly intuitive [35]. Thus, solutions for this have involved a suite of different approaches including cluster analysis [33], support vector machine classification models [23] and artificial neural networks [27].

In 2008, Wilson et al. [48] put forward a concept for a particular sensor configuration within a tag that they called the ‘Daily Diary’ (DD), where analysis yielded value well beyond the simple mathematical sum of its individual parts [48]. Specifically, the suggestion advocated the combination of tri-axial accelerometers, tri-axial magnetometers, and pressure and speed transducers together with environmental sensors such as temperature, light and humidity. The theory was that this constellation of sensors, sampled at infra-second rates, would allow tag users to be able to derive four key elements of animal ecology seamlessly. These are: (1) animal trajectory, and therefore position [45] (2) animal behaviour (Shepard, [48]) (3) energy expenditure [49] and (4) the environmental conditions to which the tag carrier is exposed [43]. Although this original work did indicate avenues by which these elements might be achieved, there was no specific suggestion of software that might actually do this. In short, currently, although some software is available to help determine some aspects of that advocated by the DD concept (e.g. [6]), there is nothing that binds the concepts together.

This paper describes the structure and functioning of a new software package (Framework4) that allows the users of smart tags to calculate all four key elements advocated by the DD and then to bind them together into one single output file so that workers can subsequently geo-reference behaviours, energy expenditures and environmental parameters to gain a more holistic picture of how animals react to and within their environment. Specifically, we introduce Framework4, a user-friendly turnkey solution for the analysis of smart sensor data. We demonstrate our software on data recorded using a DD, however, the software can be applied to any data formatted to our input specifications (see Section 2.1). Using our system, we can obtain seamless animal behaviour, animal trajectory, energy expenditure, and environmental conditions, all within one application and export them in one merged data file. Our solution requires no knowledge of the underlying processes utilised in the software, or any advanced mathematical and computing skill sets. Our application has been produced with the end-user in mind, utilising wizards and graphical user interfaces wherever possible. We hope this software will assist in the understanding of wild animal ecology, providing new insights as a result of advanced computing knowledge.

## Implementation

Framework4 has been implemented through a five year long collaboration with the Bioscience and Computer Science departments at Swansea University. The result is a software package for the Microsoft Windows operating system for analysing smart sensor data. We create a desktop application as it allows us to handle large data files effectively (tested on over 5 million data samples) on standard computing equipment. Alternatively, utilising a web application would allow access from anywhere on any platform with an active internet connection and a web browser, however, this entails long waiting times while data sets upload, resulting in a reduced ability to handle larger files. Utilising a desktop application, we directly communicate with the CPU for efficient data handling, and the GPU for visualisation purposes. Our tool can be used in the field, in remote areas during deployment where there is often no internet access. Each of the software features and the underlying methods by which they operate is now discussed. Design choices have been made at each stage in order to assist the user in their tasks, for example, making use of wizards to lead the user through various processing tasks.

### Loading data

The software supports two file formats; comma separated values (CSV) and tab delimited formats. These are two of the standard file formats for storing tabular data in text format and are common outputs from commercial smart sensor tags. The DD exports its data in a binary format which gets segmented into multiple files and converted to tabular delimitated format post-deployment. Framework4 loads and operates on the individual tabulated files.

We incorporate an import wizard in the application to import data files. Here, the user can specify the names and data types of each data attribute, which are used as a reference to them throughout the application to allow clear identification.

#### Derivation of animal trajectory by dead-reckoning

The way in which animals use the environment is fundamental to understanding their behavioural ecology [7] and, as such, many different systems have been developed to examine animal movements (see e.g. [42]). GPS has been popular for animal movement. However, fine infra-second scale behaviour cannot be obtained [3]. A relatively recent addition to the field which can achieve this is dead-reckoning [48], this has particular value in not being dependent on transmission technology. However location cannot be obtained accurately from inertial sensors due to error accumulation. What we achieve in this software is a framework that allows researchers to experiment with location fixing data channels combined with motion channels to provide corrected dead reckoning. It is not the goal of this paper to validate one method over another see Bidder et al. [3], but rather to provide the framework where animal paths can be integrated from one or a mix of data channels.

Dead-reckoning operates on the basis that the position of an animal at any time ‘*t*’ can be derived knowing the position of the animal at a previous time ‘*t-1*’ and the distance and heading taken between the two time intervals. Dead-reckoning has received little interest until now, partially because early systems for dead-reckoning were crude [20, 47, 50] but with the development of sensors and techniques, headings can now be computed to within 1° utilising accelerometer and magnetometer sensors [9, 48] by measuring the earths’ magnetic field.

The earth’s magnetic field is constructed of field lines approximating a magnetic dipole. Each field line originates at a point near the magnetic South Pole and terminates at a point near the magnetic North Pole. Measuring the strength and direction of the field lines using a tri-axial magnetometer can obtain a relative compass heading in respect to magnetic north. The relationship between magnetic north and geographic north is defined by a declination angle which varies across the earth’s surface and with time. The angle of declination can be obtained from a reference table provided by the National Geophysical Data Center (http://www.ngdc.noaa.gov/geomag-web/). Applying the declination angle to the magnetic north heading obtains a geographic heading.

Many algorithms have been introduced to this end for dead-reckoning (e.g. [21, 25, 45]) to provide new insights into animal movement. These methods have been made available to the research community through statistical software packages. Narazaki and Shiomi 2010 [26] introduced ‘ThreeD_path’, a library for the Matlab statistical package based on the dead-reckoning algorithm by Johnson and Tyack [21] for reconstructing 3-D paths. More recently, Battaile [2] created the ‘TrackReconstruction’ R package to perform dead-reckoning and enable visualization of the derived trajectories. Both of these packages hold value for those with experience with the associated statistical programming languages, allowing direct manipulation of the methods used and data supplied. However, for those without any background with these applications, the learning curve can be steep, and may appear non-trivial to those with limited experience. We provide a user-friendly protocol which requires no programming experience, allowing the user to see and access the underlying derived data at each step of the dead-reckoning procedure to provide data transparency. Furthermore, the dead-reckoning aspect is tied in closely with behaviour analysis functionality which is not offered in any existing tools.

**Fig. 1:**
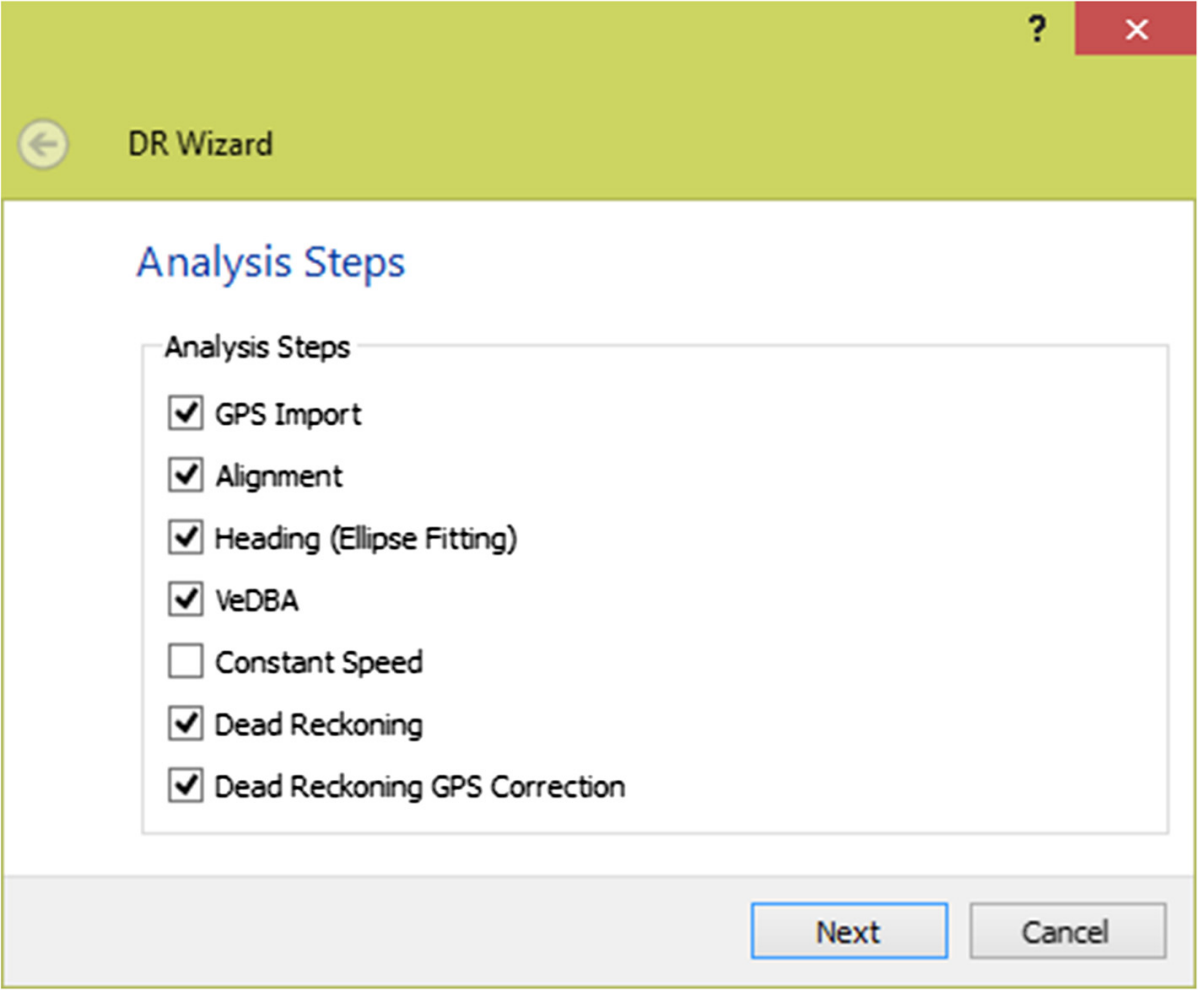
Dead-reckoning wizard - The Dead-reckoning wizard features a number of modular classification steps. These are; GPS Import, Alignment, Heading, VeDBA, Constant Speed, Dead-reckoning, and Dead-reckoning with GPS Correction

In Framework4, we introduce a user-friendly wizard for performing dead-reckoning on data with tri-axial magnetometer and tri-axial accelerometer components (Fig. 1) accessible via the ‘tools’ menu. Dead-reckoning is subject to cumulative errors and, as the heading and speed are estimates, any systematic deviations from the actual heading and speed will lead to increasing errors. To assist in reducing such errors, we incorporate the corrected dead-reckoned approach described in Bidder et al. [3] which utilises positional information (i.e. GPS fixes) and applies them as a ground truth position to force the dead-reckoned path to go through them. This resets any cumulative error but does not factor in any errors associated with obtaining the ground truth position (i.e. GPS errors). We use GPS in our examples, although the ground truth positioning data can come from any source, and therefore future technological developments in this area can be integrated into the software without any changes.

Each of the components of the dead-reckoning wizard are modular so that the user can select which analysis steps are required. The steps are; (i) GPS import for synchronising time-stamped GPS (or similar) data with the original data file (ii) alignment correction for the accelerometer and magnetometer coordinate frames (iii) heading derivation from the magnetometer channels (iv) speed derivation via a proxy derived from acceleration for obtaining an estimate of the speed, or constant speed options (vi) dead-reckoning to combine the heading and speed information to derive an animal trajectory and (vii) dead-reckoning correction using external positioning information to eliminate drift in the final dead-reckoned path. We refer the reader to Bidder et al. [3] for the full methods associated to each component of the process. We now briefly introduce these in turn, detailing the software front-end.

#### GPS import

It is often the case that positional information is recorded from separate data sources to the rest of the data. For this reason we can import positional data (e.g. GPS) from separate data files and merge it back into the complete data set, although both are assumed time-synchronised. The same import wizard is used as in the file importer to import the geo-referenced data. The user imports the data, then selects the relevant time fields in both the data sets (i.e. day, hours, minutes, seconds, and milliseconds). The wizard matches the time index columns and appends the additional data fields wherever a matching data item with the same time stamp is found. Where no aligning row exists, null values are used, which are ignored during the dead-reckoning procedure.

#### Alignment

**Fig. 2:**
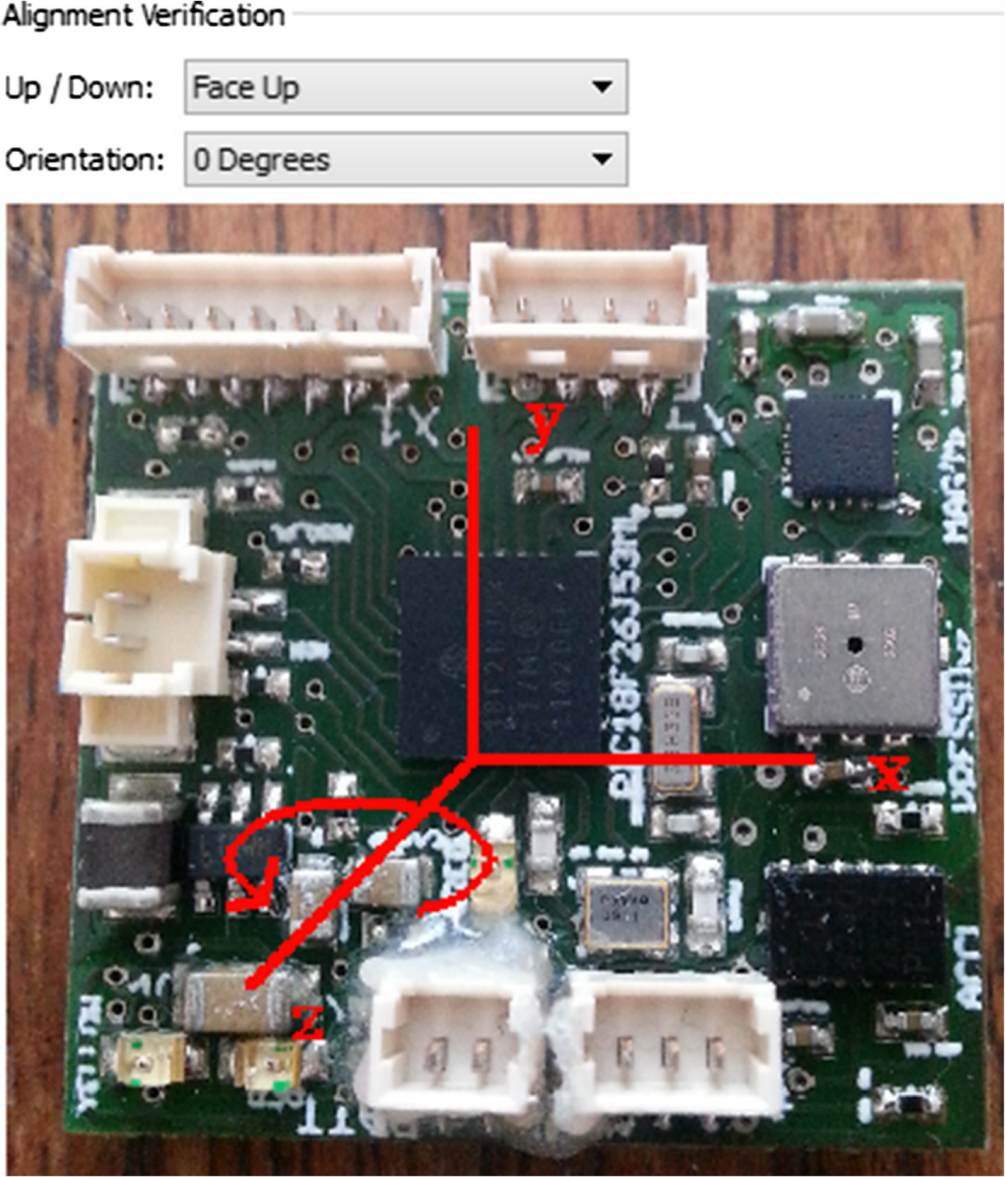
Sensor alignment correction - This image shows the alignment correction step. An image of the sensor board is updated to show the alignment selected

Alignment of the accelerometer and magnetometer coordinate systems is vital for computing heading as rotational information derived from the accelerometer channels is applied it to the magnetometer channels. Within the DD system, the coordinate frames of both sensors are not aligned (i.e. pointing in the same directions), therefore adjustment must take place. We advise users to check their sensor documentation or contact the system manufactures for this information. Data can be transformed internally using our advanced menu to select the transformations which must take place. Framework4 offers a pre-specified transformation for the DD system. In addition to this, to compute the correct pitch and roll along with device heading, the orientation of the device on the attached body must be known and corrected. To deal with this, the program asks the user to specify the orientation of the device. As the user selects different orientations, an image of the coordinate frames is updated appropriately (Fig. 2). After the correct settings have been chosen, the sensor attributes are transposed ready to calculate the heading.

#### Heading

Deriving the heading necessitates that the data attributes be sanitised via; magnetometer calibration, pitch and roll computation, coordinate frame adjustments, and finally the heading derivation. These processes are executed in the background across separate pages of the wizard and the user is exposed to the parameters and results which allow them to select the correct attributes and associated settings for each stage in the wizard. For clarity we now briefly introduce the methods for each process.

#### Ellipse fitting

The measurements obtained by commercial magnetometer sensors are corrupted by several errors. Proper calibration of the magnetometer is required to obtain high accuracy heading measurements. Inconsistencies are usually introduced by instrumental errors, such as scale factors, non-orthogonality between axes, offsets and magnetic deviations caused by perturbations and interference with the magnetic field lines.

Magnetic measurements are subject to sources of error primarily caused by hard iron and soft iron deposits acting on the magnetic field. Rotating a magnetometer around 360 degrees in all orientations under no sources of error should produce a perfect sphere centred on the origin. Hard iron effects are created by objects which produce a magnetic field with a constant bias in the output, resulting in a sphere displaced from the origin. Soft iron deposits are caused by ferrous materials which are more permissive to the magnetic field influencing the magnetic field as it passes through, via distortion or stretching. This distorts the sphere into an ellipse as hard iron errors are independent of the orientation of the device and can therefore occur across the sphere. Hard iron distortions are caused by metals such as nickel and iron and commonly have a much larger contribution towards the total error [8].

We utilise the state-of-the-art error model presented by Renaudin et al. [30] consisting of an ellipsoid-fitting algorithm based on an adaptive least squares estimator which calibrates the magnetometer readings for both instrumental errors and magnetic deviations. Prior to deployment the user is required to reposition the device at a number of orientations to obtain a complete range of values which can then be fitted to an ellipse and reformed to a uniform sphere. The user selects the magnetometer data channels which are then used to compute the correction matrix. The user can view and adjust the given matrix which can be exported and applied to other data sets from the same deployment.

#### Pitch and roll computation

In order to determine the heading of a device affixed to an animal, the magnetometer should ideally have an *x y* plane that is parallel to the earth’s surface, something that is not always possible given the mounting position and uncertainty of animal behaviour. Tilt correction for pitch and roll is computed using the static acceleration derived by passing a windowed moving average over the acceleration axes [34]. Shepard et al. [34] provide guidelines on selecting an appropriate window size. From the static acceleration the pitch and roll can be computed.

The user selects the accelerometer attributes, along with the window size to use for deriving the pitch and roll. A time-series graph shows a preview of each of the derived angles. The nature of the software allows the user to experiment with different window sizes to see the direct impact of different parameters on the resulting pitch and roll before continuing.

#### Coordinate frame adjustment

Device attitude via pitch and roll can be used to rotate the magnetometer measurements to bring them back level with the earth’s surface. Within this step, the user selects the pitch and roll channels. This allows the user to select non-derived values, for example pitch and roll, from a gyroscope for this purpose otherwise the pitch and roll from Section 2.2.3.2 should be selected. The user clicks compute and can preview the rotated magnetometer channels in a time-series graph view.

#### Heading derivation

The compass heading (H) with respect to magnetic north is determined using the *x* (*m*_*x*_) and *y* (*m*_*y*_) tilt- and error-corrected magnetometer components utilising;

$$ H=\left( arctan\left({m}_y\ / - {m}_x\right)\right) \bullet \frac{180}{\pi } $$

In this step the user selects the adjusted magnetometer components and clicks compute. A preview of the derived heading is displayed for verification.

#### Deriving speed

Dynamic acceleration [49] is argued as an good measure for predicting speed in terrestrial animals [4] and has indeed been found an effective proxy for speed in 10 disparate species including geese, armadillos, penguins, skunks, ducks, beavers, cormorants and humans, during terrestrial locomotion [4]. However, the vectorial sum of the dynamic acceleration (VeDBA) appears more robust than the overall dynamic body acceleration (ODBA) in this context since it copes better than ODBA to variability in substrate [5]. Framework4 calculates VeDBA using;

$$ VeDBA = \sqrt{D{A}_x^2+D{A}_y^2+D{A}_z^2} $$

where *DA*_*x*_, *DA*_*y*_ and *DA*_*z*_ are the dynamic acceleration values derived by taking the absolute values of running means of the raw acceleration values of each of the 3 orthogonal measurement axes from the corresponding raw acceleration values. In this step, the user specifies a window size to use to derive the dynamic acceleration component (see Section 2.2.3.2). Animal speed (*s*) can then be computed by applying a speed coefficient m and adding a constant c to the VeDBA value. The speed coefficient and offset is adjusted in the dead-reckoning wizard page. In addition, we expose a threshold *t* whereby, if the VeDBA falls below this value, the VeDBA is set to zero.

$$ s = \left\{\begin{array}{c}\hfill \left( VeDBA\bullet m\right)+c\kern3em  if\  VeDBA>t\hfill \\ {}\hfill 0\kern10.75em  else\kern1.75em \hfill \end{array}\right. $$

For volant to correspond to the speed of the animal, invalidating VeDBA in this context. Until a satisfactory measure of speed is derived (such as a pitot tube), we suggest using the constant speed option, and correct this later using positional information (like GPS).

#### Dead-reckoning

Dead-reckoning combines speed and heading to compute a trajectory for the given data. There are a number of parameters which must be defined first by the user. These are; (i) an initial start position defining the point where the path starts (if GPS data is given, the start coordinates are taken from this), (ii) a number of speed parameters for the VeDBA threshold and speed coefficients (see Bidder et al. 2012 [3–5]) and (iii) a heading offset corresponding to the declination angle obtained from the NGDC website (previous). The computed path is shown alongside in a map below the parameters so that the user can interactively adjust settings and see the result on the generated path.

To compute the path, the speed (*s*) obtained from Section 2.2.4 must be converted to radial distance in terms of the radius of the earth R (6.371 x10^6^ m). This is calculated to obtain (q) as below.

$$ q=\frac{s}{R} $$

The Latitude and Longitude at time T_i_ (where T_0_ is equal to the starting point of the track) can be calculated as follows, using the previously converted speed (*q*), and heading (*H*).

$$ La{t}_i=\mathrm{asin}\left( \sin\ La{t}_{i-1}\bullet \cos\ q+ \cos\ La{t}_{i-1} \bullet \sin\ q \bullet \cos\ H\right) $$

$$ Lo{n}_i=Lo{n}_{i-1}+ atan2\left(\Big(\  \sin\ H \bullet \sin\ q \bullet \cos\ La{t}_{i-1}\right),\ \left( \cos\ q - \sin\ La{t}_{i-1}\bullet \sin\ La{t}_i\right)\Big) $$

The complete set of latitude and longitude points defines the trajectory of the animal movement across the earth’s surface. In future work we wish to also derive vertical movement using the pressure sensor. The trajectory columns are appended to the data set once ‘next’ is pressed.

#### Dead-reckoning with position correction

The dead-reckoning solution can be subject to cumulative errors from the estimates of derived heading and speed. Even small, but systematic, errors in these channels will accumulate over time and thus can increase the resulting error correspondingly.

To overcome these problems, the program utilises a dead-reckoning correction algorithm to correct the heading and speed of the obtained dead-reckoned trajectory using aligned positional trajectory information. This resets the accumulated error at each given position.

Bidder et al. [3] correct the heading and speed by adjusting the length and heading of the dead-reckoned path until they align to the same positions along each position fix.

The heading (*θ*) between two points (*Lat*_0_, *Long*_0_) *and* (*Lat*_1_, *Long*_1_) is calculated as so:

$$ TracjectoryHeading\left(\theta \right)= arctan\Big(\frac{ \cos \left(La{t}_0\right)* \sin \left(La{t}_1\right)- \sin \left(La{t}_0\right)* \cos \left(La{t}_1\right) \cos \left(Lon{g}_1 - Lon{g}_0\right)}{ \sin \left(Lon{g}_1 - Lon{g}_0\right)* \cos \left(La{t}_1\right)} $$

The distance (*d*) between two points (*Lat*_0_, *Long*_0_) *and* (*Lat*_1_, *Long*_1_) is calculated as so:

$$ latitudeDistance\ (latD)=La{t}_1 - La{t}_0 $$

$$ longDistance\ (longD)=\kern0.5em Lon{g}_1 - Lon{g}_0 $$

$$ arcDist = \sin \left(\frac{latD}{2}\right)* \sin \left(\frac{latD}{2}\right)+ \sin \left(\frac{longD}{2}\right)* \sin \left(\frac{longD}{2}\right)* \cos \left(La{t}_0\right)* \cos \left(La{t}_1\right) $$

$$ TrajectoryDistance(d)=R*2* \arctan \left(\frac{\sqrt{arcDist}}{\ \sqrt{1- arcDist}\Big)}\right) $$

The headings and speed between fixes can be adjusted to those of the positional fixes and iteratively adjusted until the dead-reckoned and ground truth positions coincide. For two sequential ground truth fixes, there are usually many dead-reckoned fixes in-between. Firstly the heading is adjusted, this consists of adding a constant heading to all the dead-reckoned headings between the ground truth fixes.

$$ headingCoefficient(hC)=\left( gpsHeading- drHeading\right) $$

A speed coefficient adjusts the speed to that between the fixes. This consists of multiplying the speed values (derived from the VeDBA) so they equal that between the ground truth fixes.

$$ speedCoefficient(sC)=\left(\frac{gpsDistance}{drDistance}\right) $$

The formulae for dead-reckoning are adjusted to generate a dead-reckoned corrected path where *q* is the original speed, and *H* is the original heading coefficient applied. The speed coefficient is multiplied by the original speed coefficient, along with an addition of the heading offset, specific for that section of the track to counter the deviations from the geographical positions.

$$ La{t}_i=\mathrm{asin}\Big( \sin\ La{t}_{i-1}\bullet \cos \left(q*sC\right)+ \cos\ La{t}_{i-1} \bullet \sin\ q \bullet \cos \left(H+hC\right) $$

$$ Lo{n}_i=Lo{n}_{i-1}+ arctan\left(\Big(\  \sin \left(H+hC\right) \bullet \sin \left(q*sC\right) \bullet \cos \left(La{t}_{i-1}\right)\right)\ / $$

$$ \left( \cos \left(q*sC\right) - \sin \left(La{t}_{i-1}\right)\bullet \sin \left(La{t}_i\right)\right)\Big) $$

Each iteration of the formula makes the path adhere more tightly to the ground truth fixes as the heading and speeds are adjusted. We allow the user to repeat the adjustment process a set amount of times or continue until the speed and heading adjustments are under a specified threshold. Once finalised the user clicks ‘finish’ where the corrected latitude and longitude components are appended to the data set.

**Fig. 3:**
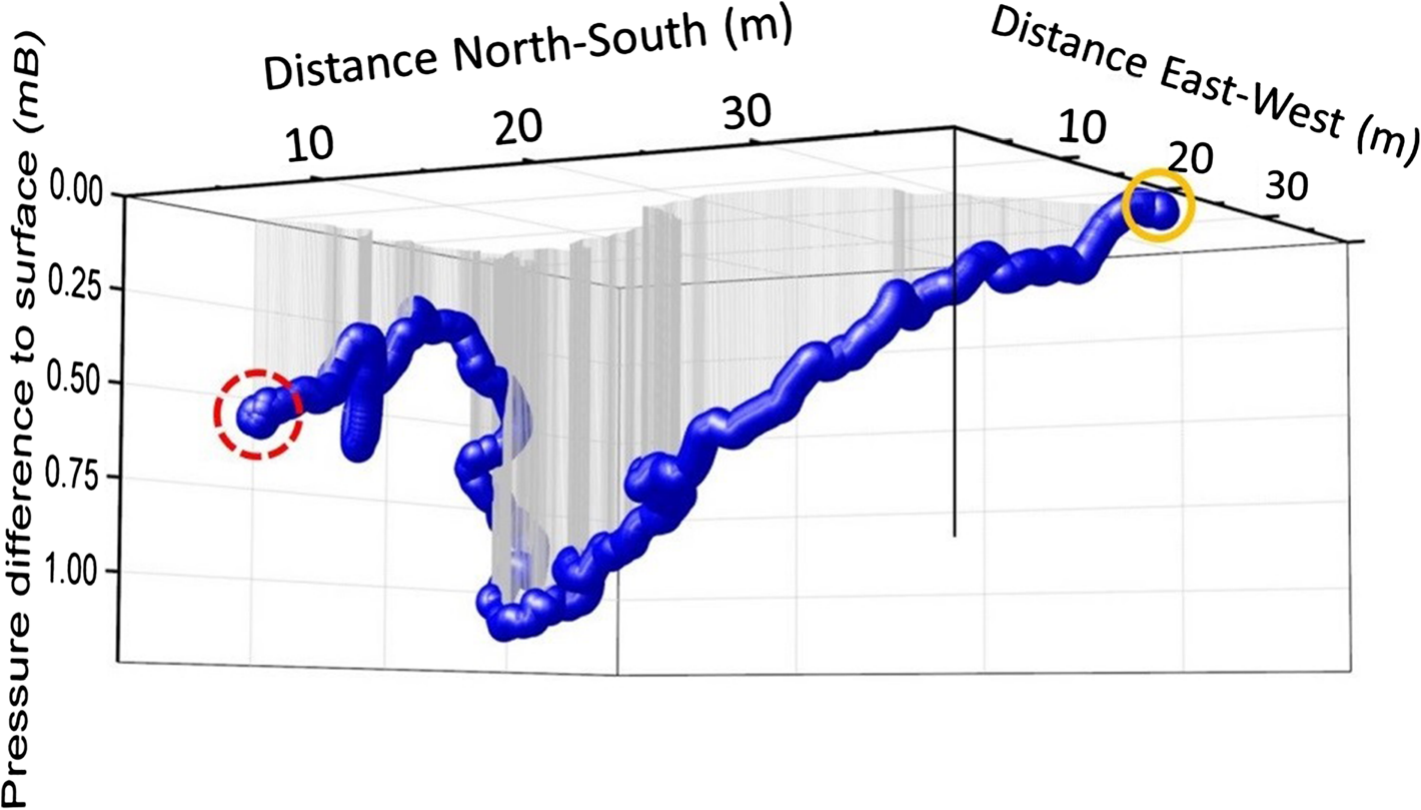
Dead-reckoned track of a European badger - Dead-reckoned track of a European badger (Meles meles) in Northern Ireland leaving its sleeping quarters (red dashed circle) and moving through the underground sett to emerge at the entrance (yellow circle). The vertical axis representing depth is shown as the pressure difference between the surface and any time underground. The reconstruction assumes that animal speed is directly proportional to VeDBA [4] underground in the same way it is on the surface. If this is not the case, the derived distances will be affected accordingly

The particular advantage of a dead-reckoned track is that it can give very fine detail in the route of an animal and do so without reference to external ground-truthing sources, although confidence in the precision of the route will inevitably decrease with increasing time between ground-truthed points (Bidder et al. [3]). Nonetheless, the approach has particular value in being able to allude to trajectories where conventional tracking methods do not work (Fig. 3).

### Derivation of animal behaviour

Extracting animal behaviour from raw sensed data (most commonly tri-axial accelerometer channels) is a time-consuming and cognitively demanding process for human analysis. Machine learning processes, namely classification, can be applied to identify and label behaviour by executing algorithms which learn from data to discover previously unknown properties [12]. The learning aspect is typically split into two categories; supervised, and unsupervised learning. Supervised learning algorithms are trained on labelled data to generalize relationships between input and output samples. Conversely, unsupervised learning algorithms operate on unlabelled data to find previously unknown structure, and domain knowledge can then be applied to match found structure to a behaviour.

Supervised techniques build a model from labelled data which generates predictions in response to new data. Traditionally, K-nearest neighbour (K-NN), support vector machines (SVM), and random forests have all been applied to accelerometer data. K-NN is a ‘lazy’ learning method which predicts class membership based on the *k* closest training examples in the feature space (e.g. [6]). The SVM algorithm finds a hyperplane which separates the feature space into the classes defined in the training set. Unseen data is assigned to a class based on the hyperplane region under which it falls (e.g. [13]). Random forests are the current state-of-the-art classification method in the data-mining community (e.g. [11]). Random forests construct many decision trees, each modelling the training set, with each tree voting for the resulting classification. A data item is assigned to the class with the most votes.

**Fig. 4:**
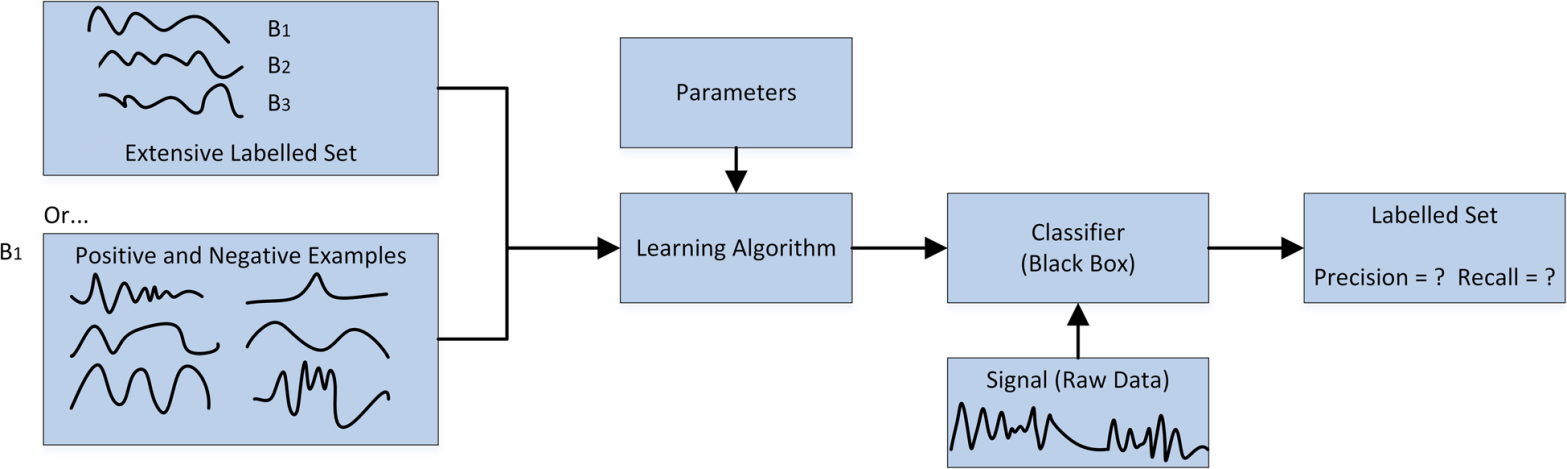
Supervised learning work-flow - An image of the typical workflow undertaken when applying supervised learning. A model learns from extensively labelled data to generalize relationships. The model operates on new unseen data to classify behaviour

Recently, there has been an interest in software packages to make supervised learning methods readily available to the movement ecology community. Ethographer [33] utilises wavelet transforms with k-means clustering to classify animal behaviour. Resheff [29] introduce AcceleRater, a web application supporting a wide array of models for supervised learning, including, K-NN, SVM, decision trees, random forests, naïve Bayes, LDA, and QDA. Figure 4 shows the typical work-flow applied when utilising supervised learning algorithms (as in the previous software applications). Firstly though, they require extensively labelled instances of behaviours. Obtaining this data is time consuming, requires domain expertise, and the undertaking of field studies to gather video-synchronised data. It is obviously not possible to obtain all such data in all cases due to environmental constraints. Secondly, the choice of algorithm and parameters provides its own class of problems. Typically, in this process, the data dimensionality is reduced to a few parameters which contain the relevant information to perform classification; feature extraction. Good classification results rely heavily on the features chosen. However, extracting a desirable feature set is considered more of an art than a science and takes a great amount of skill along with trial and error [36]. Once the data is classified, if the accuracy is less than desired, decisions must be made as to whether it is useful to invest more time creating additional training input, modify the parameters, or use a different learning algorithm. It is not obvious what the next best step to take is without sufficient knowledge of the underlying algorithms.

#### Framework4’s approach to behavioural identification

In this section we introduce the behaviour identification functionality of Framework4. Unlike the previous methods, we consider classification as an extended form of search [39]. Providing just one positive example of a behaviour, the user searches for matching behaviours and sorts them into the correct classification group. Complicated parameters are avoided by utilising interactive visual interfaces which draw from the domain expertise when selecting matching behaviours in the input signal. A feedback loop is incorporated such that the precision and recall can be boosted by applying the user’s subject knowledge. These features overcome the disadvantages of machine learning and provide a working solution that can cope with large complex data sets, a vital element given increasing sampling rates (e.g. 10 channels, each at 40 Hz).

The result is a system which supports the manual labelling of animal behaviour complimented with a user-driven approach for the semi-automatic classification of animal behaviour, requiring one instance of behaviour for the matching process to take place.

**Fig. 5:**
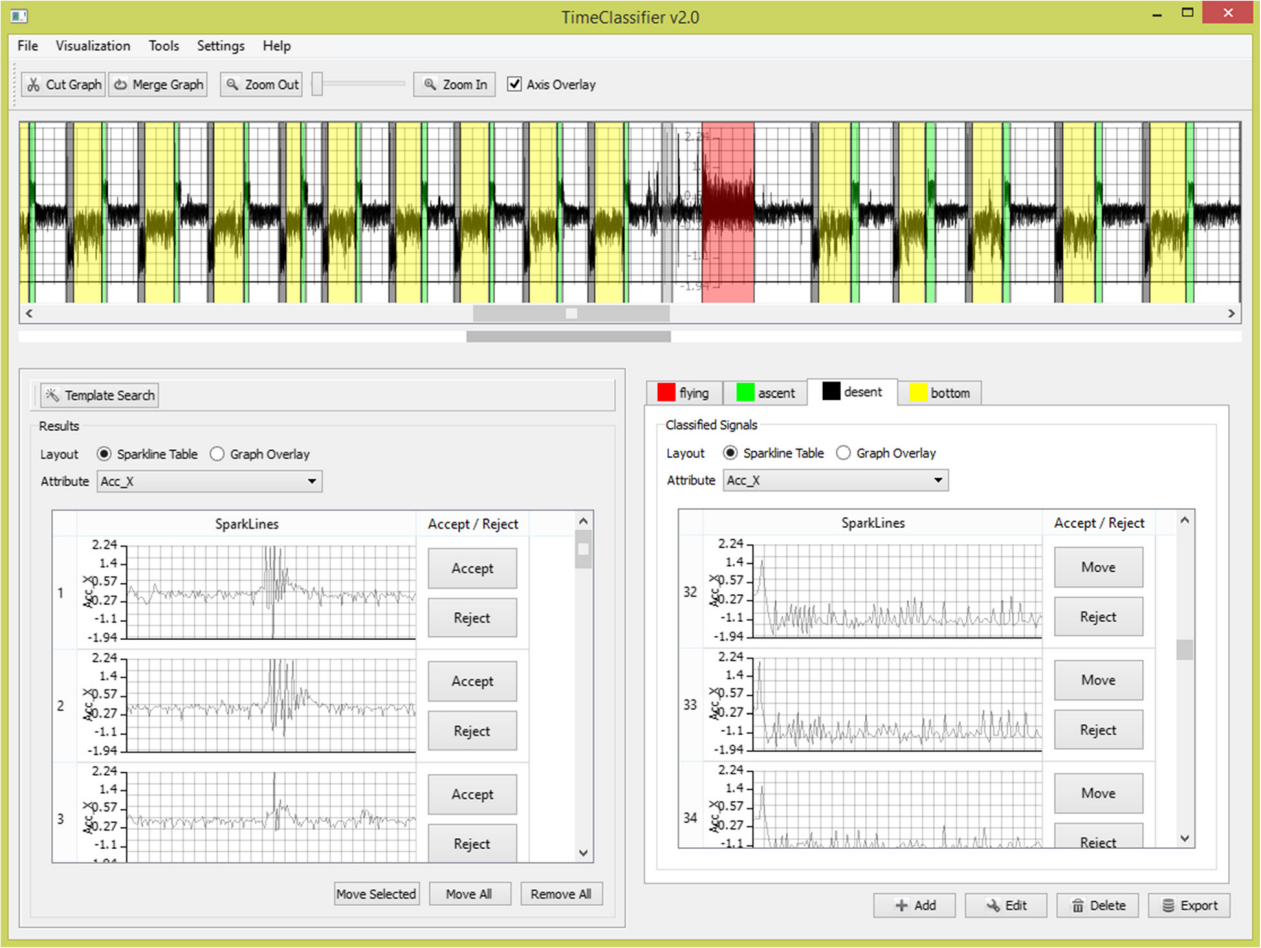
Overview of the FRAMEWORK4 user interface - An image of an overview of the user interface provided in FRAMEWORK4. In the top there is a stacked time-series graph with labelled behaviours overlaid as transparent windows. The search widget (bottom left), allows the user to search for behaviours and filter through found instances by accepting and rejecting. Accepted results are moved into the classification widget (bottom right). This view encapsulates the behaviour groups and associated classified instances

The user interface is split into three components (see Fig. 5 for overview). This consists of the data view at the top, being composed of the data in a stacked time-series graph format. Coloured segments overlaid on the graph indicate classified animal behaviour. A search panel is located in the bottom left, within which the user can perform searches on the data utilizing the template search wizard. Results are shown in this panel for the user to test, reject or accept results before moving to the appropriate classification in the bottom right panel, where the classification widget is situated. Classified behaviours are shown to the user in this tabulated panel. Each tab represents a separate behaviour and contains visualizations of the corresponding set of classified instances. The colours assigned to each tab correspond to those overlaid transparently on the time-series graph.

The components of our system and each of the processes towards classifying animal behaviour are split into five steps; (1) the user must select a behaviour to find in the data set, (2) matching is performed to find similar regions across the series, (3) A classification wizard allows the user to apply their knowledge to extract matches, (4) Extracted behaviours are presented to the user, (5) The user can improve the accuracy of their results by applying a feedback loop, (6) The classified results are displayed.

#### Behaviour selection

The first step in our system is for the user to select a behaviour to classify in the data. There are two methods for this in the application. Firstly, query-by-example, and secondly selecting previously saved behaviour instances from the template database.

Query-by-example allows the direct selection of animal behaviour by drawing a window across the time-series encapsulating a subset of data exemplifying the behaviour the user wishes to search for. After selection, a dialog is then displayed where the user can select the data attributes to utilize for the template. Any data attribute can be used for searching throughout the system (not just the accelerometer component). For example, the magnetometer attribute is useful for finding thermalling cycles in condors, while the pressure component can indicate diving cycles in aquatic species.

Behaviour templates used in the system are stored in a database back-end for future use. This is particularly useful because assigned behaviours can be used to search other files. The database can be set up to deal with behaviour templates assigned to classes of animals. The user can query for all patterns present for a specific animal or select an existing behaviour template previously saved in the database by navigating to the animal of interest and then selecting the appropriate behaviour template.

The signal may be resampled to capture events at different frequencies as some behaviours occur at different speeds, for example running. To capture these events independently of the time duration, we can store and search for the signal at different time-intervals using resampling. Re-sampling is used by specifying an irrational factor consisting of an interpolation factor (amount to up-sample by) and a decimation factor (amount to down-sample by)e.g. resampling by 1 / 2 will half the sampling rate, while resampling by 2 / 1 will double the duration.

#### Template matching

Supervised machine learning techniques rely on large bodies of labelled data. Such extensively labelled data sets do not exist in our application area. An alternative solution is to consider classification as an extended form of search. That is, to search for matching behaviours, then sort matches into the correct classification group, or reject them. Template matching is a process for determining the presence of a known waveform in a larger dataset. In essence, this works by sliding the specified template across the data set, computing the similarity of the template at each position in the data series corresponding to the concordance in fit between template and the sample at the position. The result is a similarity value at each position in the time-series. This allows the user to select a single positive example of a behaviour and search for all occurrences of it in the data.

A distance measure is used to determine a quantitative value corresponding to similarity or dissimilarity between time-series. Correlation is the optimal technique for detecting a known waveform in random noise [36]. In signal processing, it is well known that Correlation has a linear complexity frequency space. We utilise correlation and a new fast normalized cross-correlation method for template matching in order to obtain results within real time (seconds) which maintains an interactive implementation. Standard cross-correlation performs matching, taking into account amplitude information, while normalized cross-correlation normalizes the template signal and current area under the template such that amplitude shifts are not taken into account. This is important when performing matching over regions where the represented waveform may be present at different orientations. Re-sampling the signal allows us to introduce time axis distortion to extract behaviours occurring at different durations. Walker et al. [39] specify the exact nature of the algorithm used behind this aspect of Framework4. It is beyond the scope of this paper to discuss the exact methods used, but rather the software functionality available to make them accessible to the research community.

#### Classification wizard

Once the template matching algorithm has been executed, the user is presented with the pattern-matching results in the classification wizard. The classification wizard is used to guide the user through refining a similarity threshold to verify matched signals. The user interactively modifies the threshold value which corresponds to the similarity of extracted matches. Matches are depicted to the user in an intuitive format with interaction to modify the result set according to the user’s domain knowledge.

**Fig. 6:**
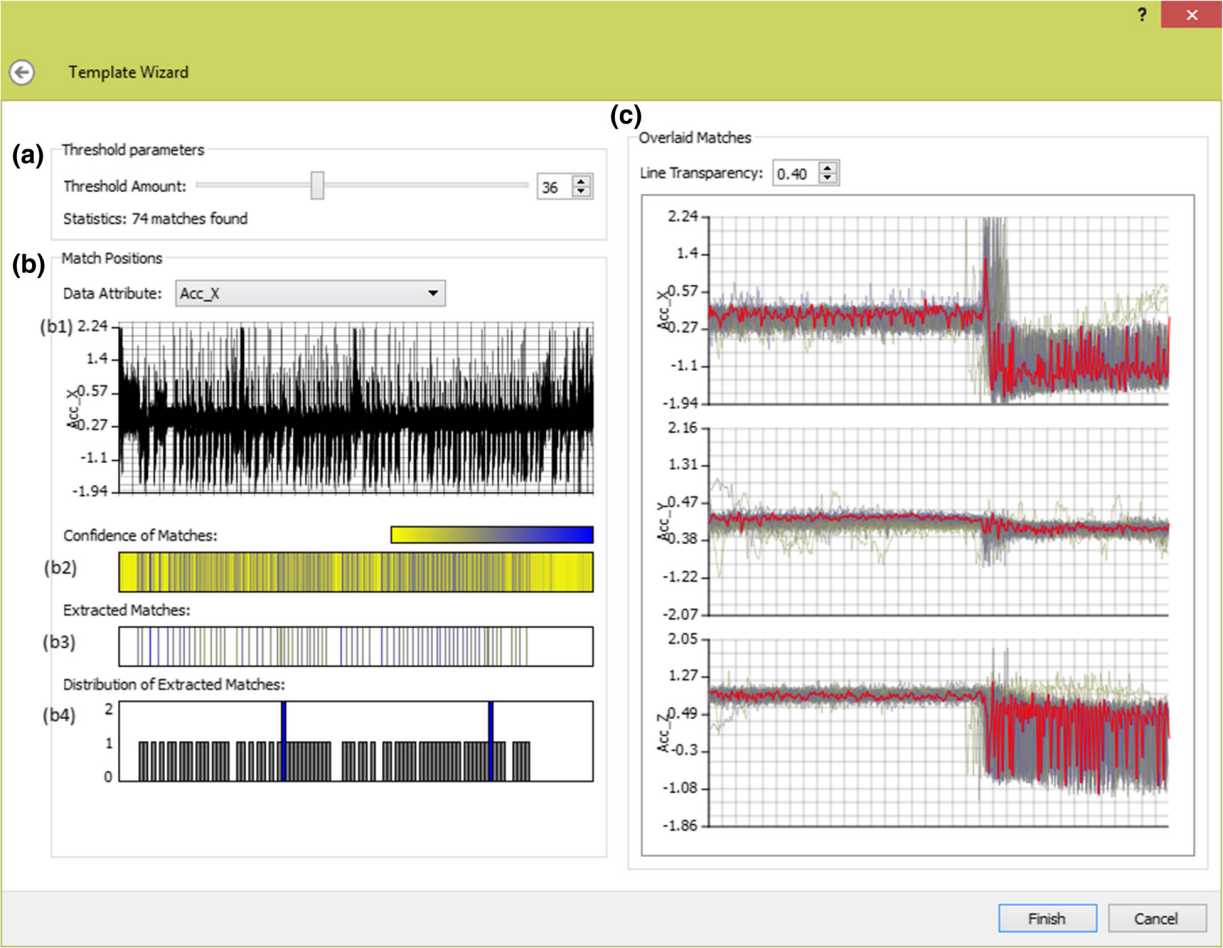
Behaviour classification wizard - This figure shows the classification wizard. **a** Illustrates our wizard parameters for dynamically adjusting the threshold. **b** Shows our density based visualizations to gain an understanding of where matches occur in the data series. **c** Shows our overlaid signals visualization of all the extracted matches in a stacked graph format, with one graph for each attribute of the template. The template signal is overlaid in red to show a direct comparison. A yellow to blue color scheme is used, yellow representing low similarity matches, while blue encodes high similarity matches

The classification wizard (Fig. 6) is used to find all instances of the specified behaviour in the data series. The aim is to then obtain an appropriate threshold value through the interaction and inspection of visualization which maximizes the number of instances found, while minimizing the number of misclassifications. The similarity threshold is represented as a percentage of the match, with one hundred percent similarity representing an exact match, while zero represents no matching features. The user needs to find an appropriate estimate value using their expert knowledge of behavioural patterns and their occurrences in the data set.

The classification wizard features two views. On the left (Fig. 6(b)) are visualisations to show where matches occur in the data series, while on the right (Fig. 6(c)), all of the extracted matches are overlaid on top of each other to show the variance between matches. The visualisations are updated as the threshold value is refined by adjusting a slider corresponding to the threshold percentage (Fig. 6(a)).

The positions where matches occur in the series are depicted using three graphical views (Fig. 6(b)), all of which are aligned with a time-series graph of the data series (Fig. 6(b1)). The confidence of a match visualization (Fig. 6(b2)) depicts a heatmap showing an overview of the pattern matching results to encode where high (blue) and low (yellow) similarity matches occur in the data series. The extracted matches view (Fig. 6(b3)) depicts where the extracted matches occur in the series and is updated as the similarity threshold is adjusted. Finally, a distribution of extracted matches (Fig. 6(b4)) utilises a histogram to show the number of matches at each position. The user may refine the result set by rejecting results by clipping rectangular regions of matching results from the data series to reject. This allows the user to reject results based on their knowledge of where they expect results to be present in the data series and the temporal trends expected.

All of the extracted matched signals are overlaid in a stacked time-series graph format, one graph for each data attribute of the pattern (Fig. 6(c)). The user can gain an overview of the general shape of the extracted signals from the graphs. This allows the verification of the shape of extracted matches as most outliers stand out immediately, not fitting into the general shape of the extracted results. The same colour-encoding scheme as the position of matches is used to encode the strength of a match. As the user adjusts the threshold, results are added or removed. The user can directly see the cause and effect of modifying the threshold on the general shape of matched signals in comparison to the template signal overlaid in red. Results can be rejected in this view by manual selection of lines on the time-series graphs. All results falling within the selection are removed from the result set. This allows the user to filter results that should not be present manually. The user continues adjusting the similarity threshold and rejecting results in the synchronised graphical views until they are satisfied with the results being extracted. The user clicks ‘finish’ and the wizard closes.

#### Results

After the user has concluded with a good threshold value, the results are extracted and added to the results widget in the bottom left of Fig. 6. The user can further inspect the results using two views. Firstly, the sparkline (embedded time-series) display, which puts the classifications in a table format, with each row corresponding to an identified instance of a behaviour visualized using a sparkline. The user can accept or reject results by selecting rows. Secondly, the overlaid plot view overlays the classified instances in a time-series graph. The user can accept or reject results by selection on the time-series. The overlaid plot view is useful where the signals shape is similar amongst results. Conversely, the sparkline display is useful where the behaviour signal varies. Matches displayed in the results view are also shown in the data view overlaid on top of the time-series graph in grey.

### Improving precision and recall

The variability and inconsistency of animal behaviour makes the automatic labelling of behaviour a challenging task. It is widely accepted in the machine learning community that achieving 100 % precision and recall is a difficult, if not impossible, task. From a movement ecology viewpoint, we aspire to achieve a close to perfect labelling of behaviour. We incorporate a feedback loop which draws from domain expertise to enhance results. Firstly, the user can provide secondary examples of a behaviour to find more behaviour instances. Secondly, the user can directly manipulate the result set to accept and reject matches. Finally, the user can manually classify behaviour.

Where the user believes the number of found instances to be low, boosting can be used to retrieve more instances. More examples of a behaviour are utilised in the template for searching. This, in effect, widens the search span to find patterns related to the secondary retrieved patterns but may not be directly related to the initial search pattern.

The results panel provides an effective means to inspect the newly found behaviour classifications. Results are accepted by moving them to an appropriate classification tab in the classification widget, or rejected by clicking the reject button. The reject button removes the result from the panel. The user should aim to keep accepting/rejecting results until this panel is empty.

We appreciate that some instances will never be identified by machine learning and may only be able to be extracted by the domain expert, be that because of a low number of instances of the behaviour, or because of the variability of the animal behaviour. We enable manual labelling in our system so that the user can manually select and classify behaviour regions. To classify a behaviour region in the data manually, the user cuts the time-series graph up into segments. Each cut contains a start and end boundary defined by that of a behaviour instance. Once a behaviour region is cut in the time-series graph, the user drags and drops the time-series segment into the appropriate classification tab. The cutting and dragging of data samples is similar to that used in video-editing software.

#### Classified results

Classified behaviours are shown in two views. Firstly, the classification widget which displays classified behaviour in a corresponding tabulated view. Secondly, classified instances are aligned and overlaid on top of time-series graphs as coloured rectangular regions identifying where in the data a match for the behaviour has occurred. Each behaviour is identified by a unique colour assigned to each classification tab in the classification widget.

**Fig. 7:**
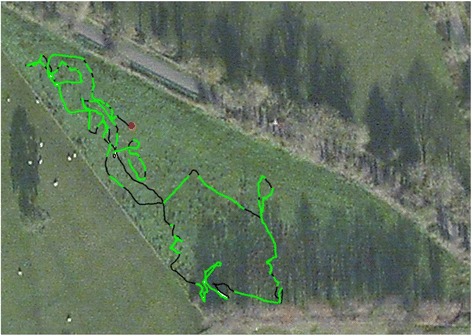
Dead-reckoned trajectory of a cow with overlaid behaviour - The dead-reckoned trajectory of a cow (Bos taurus) in a field in Northern Ireland over 2 h, colour-coded according to different activities – green = grazing, black = walking, red = lying down

A typical output of this process is that, not only can animal behaviour be classified with respect to time, but that the occurrence of different behaviours can be represented on GPS-enabled dead-reckoned animal tracks in an obvious colour scheme (Fig. 7).

### Derivation of animal energy expenditure

**Fig. 8:**
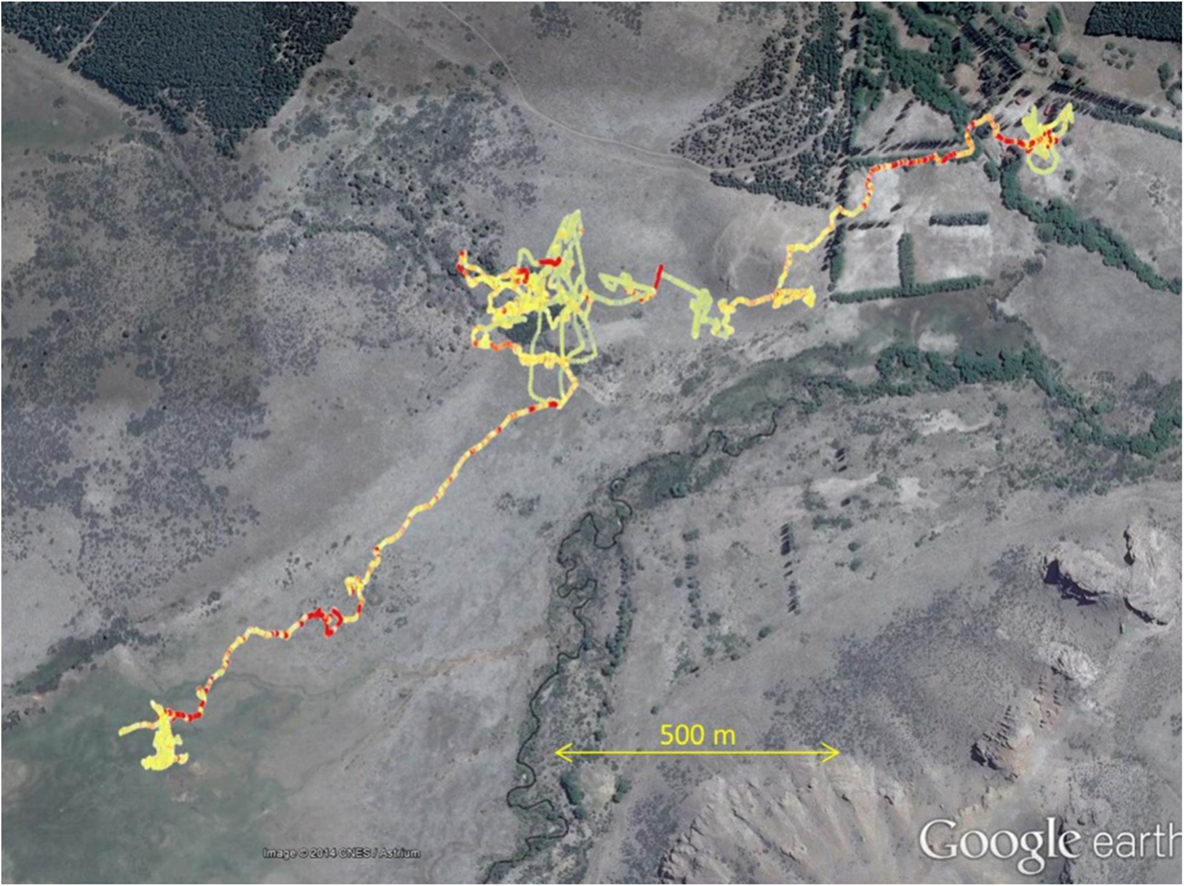
Dead-reckoned trajectory of a sheep with overlaid VeDBA - The dead-reckoned trajectory of a sheep (Ovis aries) in Argentina over 14 h showing how VeDBA, a metric that correlates linearly with metabolic rate, (ranging from pale green [low values] through yellow to red [high values] varies with location and track tortuosity. Note how higher track tortuosity is generally linked to lower VeDBA

Since the suggestion by Wilson et al. [49] that dynamic body acceleration could be used as a proxy for VO_2_, there have been a number of studies that have confirmed its utility in species ranging from shellfish, through fish, amphibia and reptiles to birds and mammals (see [17] for review). Two measures have been used, Overall Dynamic Body Acceleration (ODBA) and Vectorial Dynamic Body Acceleration (VeDBA), which are essentially equivalent in terms of their power to predict VO_2_ [28] although VeDBA has more utility for predicting speed [4]. Framework 4 uses VeDBA (see section 3.3.4 for calculation) as a proxy for VO_2_ [16] although it should be noted that dynamic acceleration-derived metrics cannot allude to metabolic costs associated with processes such as specific dynamic action and non-shivering thermogenesis [14] so users should be cognisant of this in considering the limitations of this approach. Plots of animal trajectory can be colour-coded according to VeDBA and thus highlight the link between VeDBA-derived metabolic power and location (Fig. 8).

#### Derivation of the physical characteristics of the environment

Many animals modulate their behaviour in the environment according to its physical characteristics. For instance, reptiles may associate with areas of high temperature or insolation to warm up [10] while many bird species are limited in their foraging capacities by light (e.g. [40]). Thus, the ability to resolve the geographic position of animals in tandem with environmental variables can help explain the incidence or emergence of particular behaviours (e.g. [44]). A key part of Framework4 is that it resolves animal space use and behaviour over time, inserting the positional and behavioural data into the original data file in columns. Given that DDs also record environmental variables, such as temperature [48], this means that these environmental variables are consequently linked temporally and spatially to the behaviour.

### Exporting data

The derived analytical attributes from the software can be outputted together into one data file. Exporting data is supported via navigation to the ‘Export’ option in the ‘File’ menu on the main tool bar. Export is undertaken in CSV format where derived attributes are appended as an additional column in the data file alongside the existing data channels. Each behaviour is assigned a unique numerical value where, if a data item falls within a labelled region, it is assigned this value.

## Results and discussion

The dead-reckoning and behaviour analysis components of Framework4 have been validated and compared with existing state of the art methods through two journal papers, in their respective fields. We refer the readers to Bidder et al. [3] for the verification of the dead-reckoning aspect of the software for animal movement tracks. The authors compare the dead-reckoning algorithm alongside the corrected dead-reckoning algorithm to geo-referenced data from video capture (using computer vision) along with time-aligned GPS, accelerometer, and magnetometer data. This is used to report several derived paths for comparison, dead-reckoned trajectories, GPS, dead-reckoning corrected using GPS, and dead-reckoning corrected using video at different sampling rates (refer to video in supplementary material). From this data, an error taxonomy model for the application of position correction is introduced.

Walker et al. [39] cover the behaviour labelling aspect of the software, presenting two case studies with domain experts applied to data obtained from turtles, and condors to determine specific behaviour events in the data. Using the software feedback loop, the experts were able to achieve 99.86 % and 100 % accuracy, respectively, in a short space of time. A direct comparison with machine learning techniques (hierarchical clustering, KNN, SVM, and random forests) is also presented using purely the algorithmic component of the software Walker et al. [39]. Framework4 achieves a faster run time and accuracy in comparison to these methods. A visual analytics user-in-the-loop approach (as shown in the case studies) further lets accuracy be boosted to 100 % using domain feedback through the software’s user interface, something which was previously not possible. Framework4 allows biologists to achieve unprecedented levels of accuracy for their smart sensor data using a real-time interactive algorithm.

## Conclusion

This is a first attempt to create a single program that explicitly links space use, movement, behaviour, and energy expenditure in free-living animals together with environmental conditions, doing so using an accessible column-separated format for ASCII-type data. Although there is appreciable room for improvement in many facets of the program at the moment, the aspiration is to progress and refine it to make it as powerful as possible and thereby provide a methodology which will enhance our understanding of the processes that affect the way that animals move within their environment. In future work, we seek to investigate segmenting movement tracks, applying vertical movement to dead-reckoning to generate three-dimensional movement traces, as well as investigate further visualisation techniques for movement data to expose patterns and trends in movement and behaviour.

### Availability and requirements

The software is freely available for download from the web address below. The website features instructional videos and documentation on using the software. As the website evolves, more documentation and features will be available. We hope this will be the foundation of a variety of software techniques for animal movement analysis.

- Project name: Framework4
- Project home page: http://www.framework4.co.uk
- Operating system(s): Microsoft Windows 7 or newer. 64bit and 32bit windows supported.
- Programming Language: C++ with Qt5.
- License: The program was developed by JSW and is owned by Swansea University. We encourage its free use, no permission or license is required. The current paper should be cited in resulting publications.
- Any restrictions to use by non-academics: None.
